# Liver-Specific *Commd1* Knockout Mice Are Susceptible to Hepatic Copper Accumulation

**DOI:** 10.1371/journal.pone.0029183

**Published:** 2011-12-22

**Authors:** Willianne I. M. Vonk, Paulina Bartuzi, Prim de Bie, Niels Kloosterhuis, Catharina G. K. Wichers, Ruud Berger, Susan Haywood, Leo W. J. Klomp, Cisca Wijmenga, Bart van de Sluis

**Affiliations:** 1 Department of Metabolic and Endocrine Diseases, University Medical Center Utrecht, and Netherlands Metabolomics Center, Utrecht, The Netherlands; 2 Complex Genetics Section, University Medical Center Utrecht, Utrecht, The Netherlands; 3 Department of Pathology and Laboratory Medicine, University Medical Center Groningen, University of Groningen, Groningen, The Netherlands; 4 Department of Veterinary Pathology, Faculty of Veterinary Science, University of Liverpool, Liverpool, United Kingdom; 5 Department of Genetics, University Medical Center Groningen, University of Groningen, Groningen, The Netherlands; Cambridge Institute for Medical Research, United Kingdom

## Abstract

Canine copper toxicosis is an autosomal recessive disorder characterized by hepatic copper accumulation resulting in liver fibrosis and eventually cirrhosis. We have identified *COMMD1* as the gene underlying copper toxicosis in Bedlington terriers. Although recent studies suggest that COMMD1 regulates hepatic copper export via an interaction with the Wilson disease protein ATP7B, its importance in hepatic copper homeostasis is ill-defined. In this study, we aimed to assess the effect of *Commd1* deficiency on hepatic copper metabolism in mice. Liver-specific *Commd1* knockout mice (*Commd1* ^Δhep^) were generated and fed either a standard or a copper-enriched diet. Copper homeostasis and liver function were determined in *Commd1* ^Δhep^ mice by biochemical and histological analyses, and compared to wild-type littermates. *Commd1* ^Δhep^ mice were viable and did not develop an overt phenotype. At six weeks, the liver copper contents was increased up to a 3-fold upon Commd1 deficiency, but declined with age to concentrations similar to those seen in controls. Interestingly, *Commd1* ^Δhep^ mice fed a copper-enriched diet progressively accumulated copper in the liver up to a 20-fold increase compared to controls. These copper levels did not result in significant induction of the copper-responsive genes metallothionein I and II, neither was there evidence of biochemical liver injury nor overt liver pathology. The biosynthesis of ceruloplasmin was clearly augmented with age in *Commd1* ^Δhep^ mice. Although COMMD1 expression is associated with changes in ATP7B protein stability, no clear correlation between Atp7b levels and copper accumulation in *Commd1* ^Δhep^ mice could be detected. Despite the absence of hepatocellular toxicity in *Commd1* ^Δhep^ mice, the changes in liver copper displayed several parallels with copper toxicosis in Bedlington terriers. Thus, these results provide the first genetic evidence for COMMD1 to play an essential role in hepatic copper homeostasis and present a valuable mouse model for further understanding of the molecular mechanisms underlying hepatic copper homeostasis.

## Introduction

As a redox catalyst, the trace element copper is essential to the well-being of all living organisms (reviewed by [1], [2], [3], [4]), in excess however, copper can be highly toxic due to its participation in the formation of reactive oxygen species (ROS). It is therefore important to maintain a strict balance between the essentiality and the toxicity of copper, and this involves a range of mechanisms mediating copper uptake, transport, storage and excretion. The importance of a balanced copper homeostasis in preventing toxicity is clearly illustrated by various inherited hepatic copper storage disorders such as Wilson disease (WD; OMIM #277900), Indian childhood cirrhosis (ICC; OMIM #215600), endemic Tyrolean infantile cirrhosis (ETIC; OMIM #215600) and idiopathic copper toxicosis (ICT; OMIM #215600). In WD, mutations in the *ATP7B* gene lead to copper accumulation in different tissues, particularly in liver and brain. The genetic defects underlying ICC, ETIC and ICT remain elusive, but the clinical manifestation of these non-Wilsonian copper storage disorders depends in most cases on an excessive dietary intake of copper [5], [6], [7].

Another well-documented copper overload disorder is copper toxicosis (CT) in Bedlington terriers. CT is an autosomal recessive disease linked to a homozygous genomic deletion, encompassing exon 2, of the *COMMD1* gene [8]. Affected dogs are characterized by hepatic copper overload, due to an inefficient copper excretion via the bile, resulting in liver fibrosis and eventually cirrhosis [9], [10]. In contrast to WD, Bedlington terriers affected with CT do not display any signs of neurological defects and have normal serum concentrations of the copper-bound ferroxidase ceruloplasmin (Cp) [10]. Although *COMMD1* has been suggested as a candidate gene for the non-Wilsonian copper storage disorders ICC, ETIC and ICT, no mutations in *COMMD1* have been identified in these patients so far [11], [12].

The 21 kDa ubiquitously expressed COMMD1 protein is considered as the prototype of the COMMD protein family, which is highly conserved between eukaryotes and in some protozoa [13], [14]. The ten COMMD family members (COMMD1 −10) share a C-terminal COMM domain of 70–85 amino acids that mediates protein-protein interactions and nuclear export of COMMD proteins [15], [16], [17]. Except for COMMD1, the functions of most of the COMMD proteins are largely unknown. Several studies on COMMD1 have provided supportive evidence of its role in copper homeostasis. First, COMMD1 interacts with the copper transporter ATP7B, and is suggested to regulate its proteolysis [18], [19], [20]. Second, down-regulation of COMMD1 expression results in increased intracellular copper concentrations both in HEK293T cells and the mouse hepatoma Hepa1-6 cells [20], [21]. Third, we recently demonstrated that the copper transporting activity of ATP7A, a copper transporter with high homology to ATP7B, is also mediated by COMMD1 expression [22]. Besides its role in copper homeostasis, COMMD1 is also implicated in several other pathways, such as sodium transport, antioxidant defense, and NF-κB and hypoxia signaling [17], [23], [24], [25], [26], [27], [28], [29], [30]. Interestingly, in contrast to dogs deficient for *COMMD1*, a genetic deletion of *Commd1* in mice results in embryonic lethality [31]. Altogether, these data illustrate that COMMD1 is a protein with a pleiotropic function, although its role in hepatic copper metabolism is still not well defined. To gain more insight into the function of COMMD1 in hepatic copper homeostasis in particular, and to circumvent the embryonic lethality of the *Commd1* knockout mice, we generated a hepatocyte-specific *Commd1* knockout mouse. Here, we provide the first genetic evidence that Commd1 is essential for hepatic copper excretion as Commd1-deficient mice show increased intrahepatic copper levels when their dietary copper intake is increased.

## Results

### Generation of a hepatocyte-specific *Commd1* knockout mouse model

**Figure 1:**
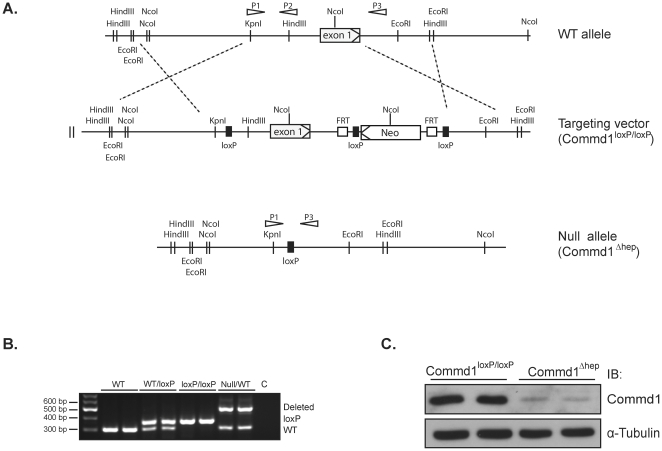
Generation of hepatocyte-specific *Commd1* knockout mouse. **A.**) Schematic representation of the *Commd1* gene-targeting strategy to generate a hepatic-specific *Commd1* knockout mouse, including a map of the *COMMD1* exon 1 allele and the targeting vector with loxP sites (solid boxes), FRT sites (open boxes), and neomycin selection gene (Neo). Homologous recombination is marked with dotted lines. Hepatocyte-specific deletion of *Commd1* was accomplished by cross-breeding of *Commd1* ^loxP/loxP^ mice with Alb-Cre mice. This resulted in the generation of *Commd1* ^Δhep^ mice (null allele). The locations of the PCR primer (P1, P2 and P3) binding sites used for genotyping are shown as open arrows. (Expected fragments: WT: 500 bp, LoxP: 350 bp, Null: 300 bp) **B.**) PCR analysis of liver tissue DNA of *Commd1* WT, WT/loxP, loxP/loxP and Null/WT mice at six weeks of age. C is a negative control (H_2_O). **C.**) Immunoblot analysis of Commd1 expression in liver tissue of *Commd1* ^loxP/loxP^ and *Commd1* ^Δhep^ mice. 30 µg tissue homogenates were analyzed by SDS-PAGE, and immunoblotted (IB) for Commd1 and α-Tubulin expression.

To circumvent embryonic lethality in Commd1-deficient mice and thus study the function of Commd1 *in vivo*, we generated a conditional *Commd1* knockout mouse. A *Commd1* targeting construct was designed to flank exon 1 of *Commd1* with loxP recombination sites by homologous recombination (Figure 1A). After confirming homologous recombination by long–range PCR (data not shown), the mice were further genotyped by multiplex PCR as described in Materials and Methods and Figure 1B. Germline deletion of *Commd1* resulted in embryonic lethality between days 9.5 and 10.5 of gestation (data not shown), similar to what we have demonstrated previously [31].

To elucidate the role of COMMD1 in hepatic copper metabolism, we deleted *Commd1* specifically in hepatocytes using the transgenic Albumin-Cre (Alb-Cre) mice (Figure S1), referred to as *Commd1* ^Δhep^ mice from here onwards. *Commd1* ^Δhep^ mice were born in the expected Mendelian frequency. The total body and liver weights of the *Commd1* ^Δhep^ mice were comparable to control littermates (*Commd1* ^loxP/loxP^) (Table S1). Immunoblot analyses of liver homogenates prepared from *Commd1* ^Δhep^ mice showed an almost complete loss of Commd1 expression relative to *Commd1* ^loxP/loxP^ mice (Figure 1C). As expected, a residual Commd1 expression was observed as the Alb-Cre transgene is selectively expressed in the parenchymal cells, but not in the non-parenchymal cells present in the liver (e.g. Kupffer, endothelial, and stellate cells) [32]. Commd1 expression in other tissues of the *Commd1* ^Δhep^ mice was unaffected (data not shown).

### Ablation of hepatic Commd1 results in elevated copper concentrations in the livers of young mice

**Figure 2:**
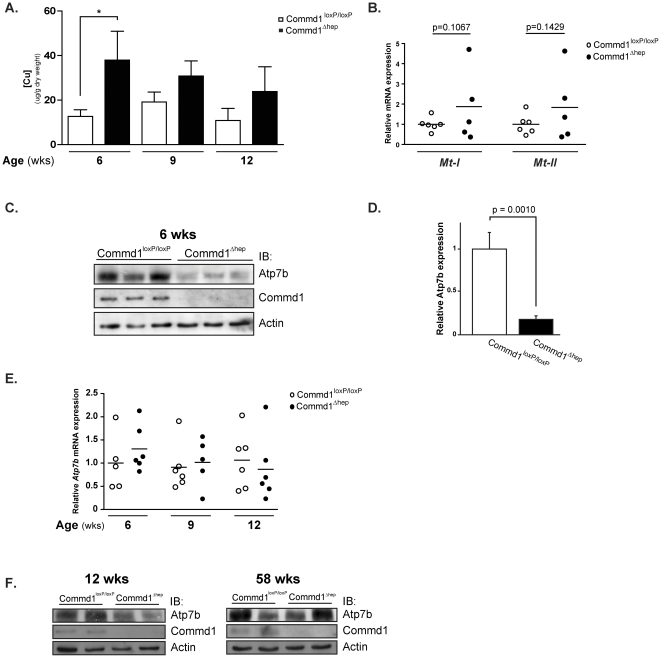
Ablation of hepatic Commd1 expression results in increased intracellular copper concentrations accompanied with decreased Atp7b expression in young mice. **A.**) Hepatic copper concentrations were measured in dried liver tissue of C*ommd1* ^loxP/loxP^ (white bars; n = 6) and *Commd1* ^Δhep^ mice (black bars; n = 5) (6, 9 and 12 weeks of age) by means of FAAS. Data are represented as the hepatic copper concentrations (µg/g dry liver weight) in all mice at indicated ages. ^*^ indicates significantly different values compared to C*ommd1* ^loxP/loxP^ mice (p<0.05). **B.**) Relative mRNA expression of metallothioneins *Mt-I* and *Mt-II* in liver tissue of C*ommd1* ^loxP/loxP^ (open dots; n = 6) and *Commd1* ^Δhep^ mice (black dots; n = 5) (six weeks of age) as determined by qPCR analysis. Expression was normalized for *β-Actin* mRNA levels, and relatively expressed to C*ommd1* ^loxP/loxP^ mice. **C.**) Immunoblot analysis of liver tissue of C*ommd1* ^loxP/loxP^ and *Commd1* ^Δhep^ mice at six weeks of age. 30 µg of liver homogenates were analyzed by SDS-PAGE, and immunoblotted (IB) for expression of Atp7b and Commd1. Actin protein expression was used as the loading control. **D.**) Densitometric quantification of Atp7b expression at six weeks of age (Figure 2C), normalized to Actin expression. Mean expression of C*ommd1* ^loxP/loxP^ mice was set at 1 ± SD. p = 0.0010. **E.**) Relative mRNA expression of *Atp7b* in liver tissue of C*ommd1* ^loxP/loxP^ (open dots; n = 5–6) and *Commd1* ^Δhep^ mice (black dots; n = 5–6) (6, 9 and 12 weeks of age) as determined by qPCR analysis. Expression was normalized for *β-Actin* mRNA levels, and relatively expressed to C*ommd1* ^loxP/loxP^ mice. **F.**) Immunoblot analysis of Atp7b expression in liver tissue of C*ommd1* ^loxP/loxP^ and *Commd1* ^Δhep^ mice at 12 and 52 weeks of age.

Since loss of COMMD1 in Bedlington terriers results in hepatic copper accumulation, we investigated the consequence of hepatic Commd1 deficiency on the amount of hepatic copper in the livers of *Commd1* ^Δhep^ mice of different ages (6, 9, 12, 34, 46 and 58 weeks; Table S1). At an age of six weeks, hepatic copper concentrations were significantly increased in *Commd1* ^Δhep^ mice compared to control animals (*Commd1* ^loxP/loxP^) (46.2±9.9 vs. 13.7±2.0 µg/g dlw, respectively; Figure 2A and Table S1). However, during adolescence, the amount of copper in the livers of *Commd1* ^Δhep^ mice declined to levels similar to those of the control mice (Figure 2A and Table S1). Although hepatic Commd1 ablation resulted in elevated hepatic copper pools, analysis of the mRNA expression of the copper-responsive genes metallothionein I and II (*Mt-I* and *Mt-II*) revealed no significant changes between six week-old *Commd1* ^Δhep^ and *Commd1* ^loxP/loxP^ mice (Figure 2B). Interestingly, the protein levels of Atp7b were markedly reduced in the livers of *Commd1* ^Δhep^ mice at this age (Figure 2C, 2D), while the *Atp7b* mRNA expression remained unaffected (Figure 2E). However, over time, Atp7b increased to levels comparable to those seen in control mice (Figure 2F), and correlated perfectly with the decline in hepatic copper concentrations in *Commd1* ^Δhep^ mice, starting at an age of nine weeks (Figure 2A and Table S1). Further, no alterations in the serum Cp activity or protein levels could be detected in six week-old *Commd1* ^Δhep^ compared to *Commd1* ^loxP/loxP^ mice in spite of the reduced Atp7b levels upon Commd1 deficiency (Table S1 and data not shown). As no differences in serum Cp activity were seen between the two groups at all studied ages (Table S1), our data imply that incorporation of copper into Cp in the trans-Golgi network is not affected by hepatic Commd1 ablation.

Despite the increased hepatic copper levels in six week-old *Commd1* ^Δhep^ mice, no overt macroscopic nor microscopic differences were identified between livers of *Commd1* ^Δhep^ and *Commd1* ^loxP/loxP^ mice (data not shown). Furthermore, copper deposits could not be visualized in *Commd1* ^Δhep^ mice livers (data not shown). Consistent with the absence of liver pathology, no differences in the liver enzyme serum levels of glutamic oxaloacetic transaminase (GOT) and glutamic pyruvic transaminase (GPT) were observed (Table S1). Taken together, these data demonstrate that ablation of hepatic *Commd1* results in a temporary copper accumulation in young mice without inducing any hepatocellular damage.

### Progressive hepatic copper accumulation in *Commd1* ^Δhep^ mice fed a high copper diet

**Figure 3:**
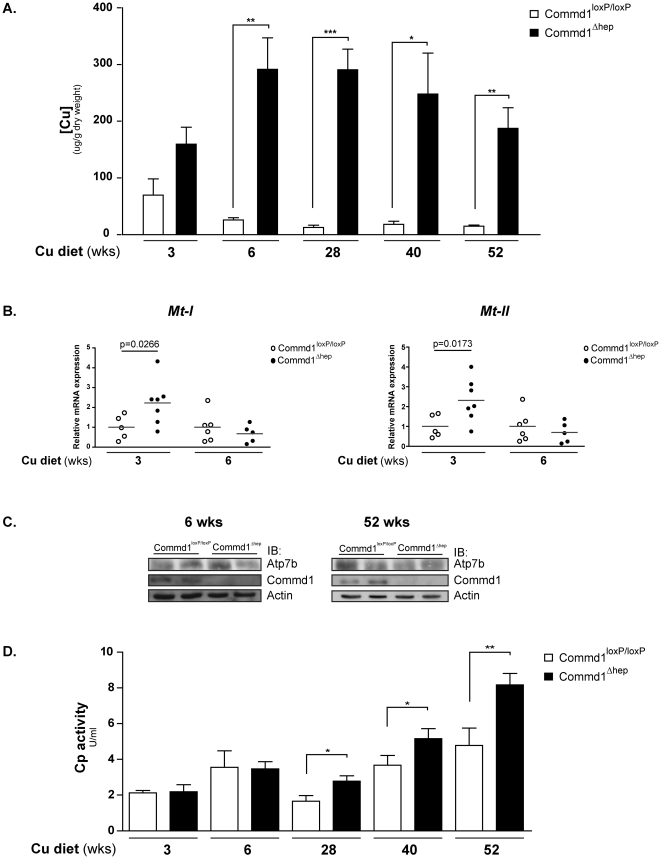
Progressive copper accumulation in livers of *Commd1* ^Δhep^ mice after copper challenging. **A.**) Hepatic copper concentrations were measured in dried liver tissue of *Commd1* ^loxP/loxP^ (white bars; n = 4–6) and *Commd1* ^Δhep^ mice (black bars, n = 5–7) (fed a copper-enriched diet for 3, 6, 28, 40 and 52 weeks) by means of FAAS. Data are represented as hepatic copper concentrations (µg/g dry liver weight). ^*^, ^**^ and ^***^ indicate significantly different values compared to *Commd1* ^loxP/loxP^ mice (^*^ p<0.05, ^**^ p<0.005, ^***^ p<0.0005). **B.**) Relative mRNA expression of metallothioneins *Mt-I* and *Mt-II* in liver tissue of *Commd1* ^loxP/loxP^ (open dots; n = 5–6) and *Commd1* ^Δhep^ mice (black dots; n = 5–7) (three and six weeks fed a copper-enriched diet) as determined by qPCR analysis. Expression was normalized for *β-Actin* mRNA levels, and relatively expressed to *Commd1* ^loxP/loxP^ mice. **C.**) Immunoblot analysis of Atp7b and Commd1 in liver tissue of *Commd1* ^loxP/loxP^ and *Commd1* ^Δhep^ mice fed a copper-enriched diet for 6 and 52 weeks. **D.**) Ceruloplasmin activity was determined in sera of *Commd1* ^loxP/loxP^ (white bars; n = 4–6) and *Commd1* ^Δhep^ mice (black bars; n = 5–7) fed a copper-enriched diet for 3, 6, 28, 40 and 52 weeks. Data are represented as serum holoceruloplasmin activity (U/ml). ^*^ and ^**^ indicate significantly different values compared to *Commd1* ^loxP/loxP^ mice (^*^ p<0.05, ^**^ p<0.01).

Since the occurrence of copper toxicosis is often dependent on dietary copper intake [5], [6], [7], [9], we challenged *Commd1* ^Δhep^ and control mice with a copper-enriched diet and followed them over time. For this, CuCl_2_ was supplemented to the drinking water to a final concentration of 6 mM (fed *ad libitum*). High dietary copper had no effect on the total body and liver weights of either *Commd1* ^loxP/loxP^ or *Commd1* ^Δhep^ mice (Table S2), but clearly affected the hepatic copper concentrations (Figure 3A and Table S2). After three weeks of high dietary copper intake, starting at an age of six weeks, Commd1 deficiency resulted in markedly raised hepatic copper relative to *Commd1* ^loxP/loxP^ mice fed a standard diet (195.8±58.9 vs. 22.6±7.9 µg/g dlw, respectively). In contrast, the hepatic copper concentrations of *Commd1* ^loxP/loxP^ mice were unaffected by the copper-enriched diet (25.8±10.7 vs. 22.6±7.9 µg/g dlw). The highest copper concentrations were measured in the livers of *Commd1* ^Δhep^ mice fed the copper-enriched diet for six weeks (338.3±82.4 vs. 11.8±6.3 µg/g dlw), which subtly declined during aging (Figure 3A and Table S2). This decline in hepatic copper was observed in both genetic groups (Figure 3A and Table S2).

Although a significant accumulation in hepatic copper was observed in *Commd1* ^Δhep^ mice fed a high copper diet, no macroscopic or microscopic alterations in their liver pathologies were identified (Figure S2 and data not shown). Neither were there differences in the enzymatic activities of serum GOT and GPT between the two groups (Table S2). Additionally, histological hepatic copper deposits were undetectable in *Commd1* ^Δhep^ mice (data not shown). It was noteworthy that mRNA expression of *Mt-I* and *Mt-II* was significantly increased in the livers of *Commd1* ^Δhep^ mice fed the copper-enriched diet for three weeks compared to controls. However, no differences in *Mt-I* and *Mt-II* expression were seen between the two genetic groups fed the copper-enriched diet for six or more weeks (Figure 3B and data not shown). Furthermore, we did not observe any differences in hepatic Atp7b levels between *Commd1* ^loxP/loxP^ and *Commd1* ^Δhep^ mice (Figure 3C). Yet, during aging, the serum Cp activity of *Commd1* ^Δhep^ mice (28, 40 and 58 weeks old) was significantly increased relative to controls (Figure 3D and Table S2).

Altogether, these results show that liver-specific Commd1-deficient mice are susceptible to progressively accumulate hepatic copper when overexposed to environmental copper. However, hepatic deletion of Commd1 does not affect the incorporation of copper into Cp.

## Discussion

Although a genomic deletion of *COMMD1* is associated with CT in Bedlington terriers, the significance of COMMD1 in mammalian copper homeostasis remains poorly defined. Here, we examined the role of COMMD1 in hepatic copper homeostasis using a liver-specific Commd1-deficient mouse model, and were able to provide substantial evidence that Commd1 plays a role in controlling copper homeostasis in hepatocytes. We demonstrated that mice deficient for hepatic Commd1 are more susceptible to hepatic copper accumulation compared to wild-type mice when their dietary copper intake is increased. A significant increase in hepatic copper concentrations was also observed in six week-old *Commd1* ^Δhep^ mice fed a standard diet, but these elevated levels declined during adolescence to concentrations similar as seen in wild-type littermates. This increase in hepatic copper in six week-old *Commd1* ^Δhep^ mice probably results from residual copper pools accumulated in the preweaning period [33], [34]. Dietary studies have not been reported in Bedlington terriers with the homozygous *COMMD1* deletion, but since most commercial dog food contains copper levels that exceed the minimum recommended daily intake [10], [35], together with the presented data, suggest that reducing the gastrointestinal copper uptake by decreasing the dietary copper content would be beneficial to the liver pathology of affected dogs.

Although our mouse model partially recapitulates the copper accumulation phenotype of Bedlington terriers affected with CT, the exact mode of COMMD1 action in regulating hepatic copper metabolism remains elusive. However, several assumptions can be drawn from our data. Similar to Bedlington terriers, hepatic Commd1 deficiency in mice does not affect the incorporation of copper into Cp by Atp7b. Importantly, probably due to the increased bioavailable hepatic copper, the biosynthesis of holoceruloplasmin was even enhanced in middle-aged *Commd1* ^Δhep^ mice fed a copper-enriched diet compared to controls. Together with the observation that the copper-induced trafficking of ATP7B to the cell periphery is unaffected in COMMD1-deficient cells [18], [20], it is tempting to speculate that, in excess copper, COMMD1 acts downstream of ATP7B and might be involved in the final step of the secretory pathway to efficiently release copper into the bile. This idea is further supported by the fact that COMMD1 partly localizes to vesicles of the endocytic pathway and cellular membranes, and shows only limited co-localization with ATP7B in HepG2 cells [18], [20]. However, COMMD1 is also implicated in regulating the protein levels of ATP7B [18], [20]. Whereas we previously demonstrated that COMMD1 expression augments the protein degradation of ATP7B *in vitro* [18], others have shown a decline in Atp7b expression after depletion of Commd1 in the mouse hepatoma Hepa1-6 cells [20]. In line with this latter observation, a marked decrease in hepatic Atp7b in six week-old *Commd1* ^Δhep^ mice was observed, and may account for the increased hepatic copper levels observed in these animals. However, no correlation was seen between the degree of copper accumulation and Atp7b levels in *Commd1* ^Δhep^ mice fed a copper-enriched diet, which argues against the role of impaired Atp7b protein stability in progressive copper accumulation in Commd1-deficient hepatocytes. Additionally, no discrepancies in Atp7b stability in primary Commd1-deficient hepatocytes compared to WT control cells were seen (data not shown). Altogether, our data indicate that COMMD1 controls hepatic copper homeostasis downstream of ATP7B and may participate in the release of copper into the bile. Further studies are however needed to complete our understanding on the molecular function of COMMD1 in hepatic copper homeostasis.

Interestingly, although *Commd1* ^Δhep^ mice fed a copper-enriched diet displayed a progressive increase in hepatic copper, no obvious liver pathology using histological analysis were seen, even after chronic exposure to high dietary copper. These data, supported by biochemical parameters and together with the observation that the mRNA expression of the copper-responsive genes *Mt-I* and *Mt-II* was only increased in mice fed a copper-enriched diet for three weeks, suggest that the accumulating copper upon *Commd1* deletion is stored safely and does not reach a threshold concentration sufficient to induce hepatocellular toxicity as seen in CT-affected Bedlington terriers and mouse models for WD [9], [10], [36]. Potentially, under these studied conditions, the levels of Mt-I and Mt-II are sufficient to chelate the elevated copper. Therefore, it would be of interest to complementary deplete *Mt-I* and *Mt-II* [37] in our hepatic-specific *Commd1* knockout mice and assess the protective role of Mt-I and Mt-II in copper toxicity in the absence of Commd1. In contrast to *Commd1* ^Δhep^ mice fed a high copper diet, which display copper concentrations of approximately 340 µg/g of dlw, CT-affected dogs with moderate to severe liver pathology show significantly more hepatic copper, often in excess of 1,000 µg/g of dlw. The reason for the interspecies differences is currently unknown and further studies are required. Of particular interest in this would be defining the degree of redundancy between the members of the Commd protein family in murine copper homeostasis, as in addition to COMMD1, COMMD2, 8 and 10 have also the ability to interact with ATP7B (Figure S3A). Importantly, these interactions are independent of COMMD1 expression (Figure S3B).

Together, our data conclusively shows that COMMD1 plays a significant role in copper homeostasis and demonstrates that hepatic copper accumulation due to loss of Commd1 is dependent on excessive dietary copper intake. Given that elevated asymptomatic hepatic copper in Atp7b deficient mice has a significant effect on different metabolic pathways, such as lipid metabolism [38], [39], [40], it would be of interest to investigate whether diet-induced copper accumulation in *Commd1* ^Δhep^ mice also affects these pathways. We believe that our *Commd1* ^Δhep^ mice represent a valuable and interesting model for further elucidating the molecular mechanism controlling hepatic copper homeostasis and to understand the role of excess copper in various metabolic pathways.

## Materials and Methods

### Generation and housing of transgenic mice

Detailed information regarding the generation of the hepatocyte-specific *Commd1* knockout mice is available in Data S1 and Figure S1. Mice were genotyped by a standard PCR method using the primers as described in Table S3, and fed *ad libitum* with a standard rodent diet containing 16.44 mg copper per kg (Special Diet Services Ltd., UK). Animals of both sexes were included in this study, and age-matched siblings were used as controls in all experiments. All animal protocols (ID 2007.III.09.123) were approved by the Institutional Animal Care and Use Committee of Utrecht University (Utrecht, the Netherlands).

### Copper treatment of mice

Starting from the age of six weeks, a subset of mice (consisting of genotypes *Commd1* ^loxP/loxP^ and *Commd1* ^Δhep^; n = 5–8) were given water supplemented with 6 mM CuCl_2_. As described previously, these mice ingested approximately 50–100 times more copper than mice fed a standard rodent diet [41].

### Tissue preparation, protein isolation and immunoblot analysis

Mice were sacrificed and tissues were rapidly isolated, frozen in liquid nitrogen, and stored at −80°C until use. Dissected tissues were homogenized in ice-cold lysis buffer (25 mM kPi buffer; pH 7.4, 0.5 M EDTA), supplemented with 100 mM PMSF and protease inhibitors (Complete; Roche, Basal, Switzerland)). After centrifugation, supernatants were used for further procedures. Protein concentrations were determined by the Bradford Protein Assay (Bio-Rad Laboratories Inc., Hercules, CA, USA).

Western blot analyses were performed using the following antibodies: rabbit-anti-COMMD1 antiserum [42], polyclonal rabbit-anti-Atp7b antiserum (kindly provided by Dr. J. Gitlin, St. Louis, MO, USA), polyclonal rabbit-anti-Actin (Sigma-Aldrich, St. Louis, MO, USA), and rabbit-anti-α-Tubulin (Abcam, Cambridge, UK). In all analyses, equal amounts of proteins were loaded on SDS-PAGE gels prior to transfer on to nitrocellulose membranes.

### RNA isolation and quantitative - RT-PCR

Total RNA was isolated from mouse liver by means of TRIZOL® (Invitrogen Life Technologies Corporation, Carlsbad, CA, USA). cDNA synthesis was performed using random hexamers and SuperScript II reverse transcriptase (Invitrogen). mRNA expression of *Mt-I*, *Mt-II* and *Atp7b* (primers previously described by Huster *et al.* [36]) was analyzed by quantitative PCR using iTaq™ SYBR®Green Supermix with ROX (Bio-Rad) and 7900 HT Fast Real-Time PCR System (Applied Biosystems, Carlsbad, CA, USA). Results were presented as relative mRNA expression, normalized to the expression of *β-Actin* (primer sequences available on request).

### Determination of hepatic copper concentrations

Liver tissues were dried at approximately 100°C until their weights were stabilized. Dried tissues were digested for 1 h in HNO_3_∶H_2_O_2_ (ratio 3∶1) at 95–100°C. After digestion, volumes were equalized and copper concentrations were determined by means of flame atomic absorption spectrometry (FAAS; Analytik Jena ContrAA® 700, Analytik Jena AG, Jena, Germany). Hepatic copper concentrations were corrected for dry liver weight (dlw) and protein concentration.

### Enzyme activity assays

Activity of the glutamic oxaloacetic transaminase (GOT) and glutamic pyruvic transaminase (GPT) were quantified in serum according to the manufacturer's protocol (Spinreact, Sant Esteve De Bas, Spain). Serum Cp activity was measured as described previously [43].

### Statistical analysis

The quantitative data in this paper is represented as means ± SEM, unless stated otherwise. Statistical evaluation was made using the Student's t-test and differences were considered to be significant at p<0.05.

Additional Materials and Methods can be found in the Data S1.

## Supporting Information

Figure S1 **Generation of hepatocyte-specific** ***Commd1*** **knockout mouse.** Schematic representation of the *Commd1* gene-targeting strategy used to generate a hepatic specific *Commd1* knockout mouse, including a map of the *COMMD1* exon1 allele, the targeting vector with loxP sites (solid boxes), FRT sites (open boxes), and neomycin selection gene (Neo). Different restriction sites are indicated and homologous recombination is marked with dotted lines. The neomycin selection cassette was deleted by crossbreed with the FLPe deleter mice, which target the FRT sequences flanking neomycin. Subsequently, hepatocytespecific deletion of *Commd1* was accomplished by crossbreed of *Commd1* ^loxP/loxP^ mice with *Alb-Cre* mice. This resulted in the generation of *Commd1* ^Δhep^ mice (null allele). The locations of the PCR primer (P1, P2 and P3) binding sites used for genotyping are shown as open arrows.
(TIF)

Figure S2 ***Commd1*** **^Δhep^ mice do not display any pathological abnormalities relative to** ***Commd1*** **^loxP/loxP^ mice.** Liver sections (4 µm) of *Commd1* ^loxP/loxP^ and *Commd1* ^Δhep^ mice fed a copper-enriched diet for 6 weeks were stained with H&E, and analyzed by light microscopy (magnification 10×).
(TIF)

Figure S3 **COMMD2, COMMD8 and COMMD10 interact with ATP7B, independently of COMMD1.** **A.**) Gluthatione-sepharose (GSH) precipitation of HEK293T cell lysates transfected with cDNA constructs encoding GST or each of the COMMD proteins fused to GST in combination with ATP7B-Flag. Precipitates were washed and separated by SDS-PAGE and immunoblotted as indicated. Input indicates direct analyses of cell lysates. **B.**) HEK293T cells expressing a stable knockdown of COMMD1 (shCOMMD1) were transfected with cDNA constructs encoding an empty vector (pEBB) or ATP7B-Flag in combination with either COMMD2, COMMD8, or COMMD10 as GST fusion proteins as indicated. HEK293T cells stably transfected with an empty shRNA vector was used as a negative control (shControl). GSH precipitation and immunoblot analysis was performed as described under S3A. Equal loading was confirmed by immunoblotting for SCHAD.
(TIF)

Table S1 **Biological parameters of Commd1^loxP/loxP^ and Commd1^Δhep^ mice fed a standard diet.**
(PDF)

Table S2 **Biological parameters of Commd1^loxP/loxP^ and Commd1^Δhep^ mice fed a high Cu diet, starting at an age of 6 weeks.**
(PDF)

Table S3 **Oligonucleotide sequences used for genotyping mice.**
(DOC)

Data S1 **Supplementary** **Materials and Methods** **.**
(DOC)
