# Supplementary figures and images for: Liver-Specific Commd1 Knockout Mice Are Susceptible to Hepatic Copper Accumulation
Source: PLoS One. 2011 Dec 22;6(12):e29183. doi: 10.1371/journal.pone.0029183 (PMC3245254; doi:10.1371/journal.pone.0029183)

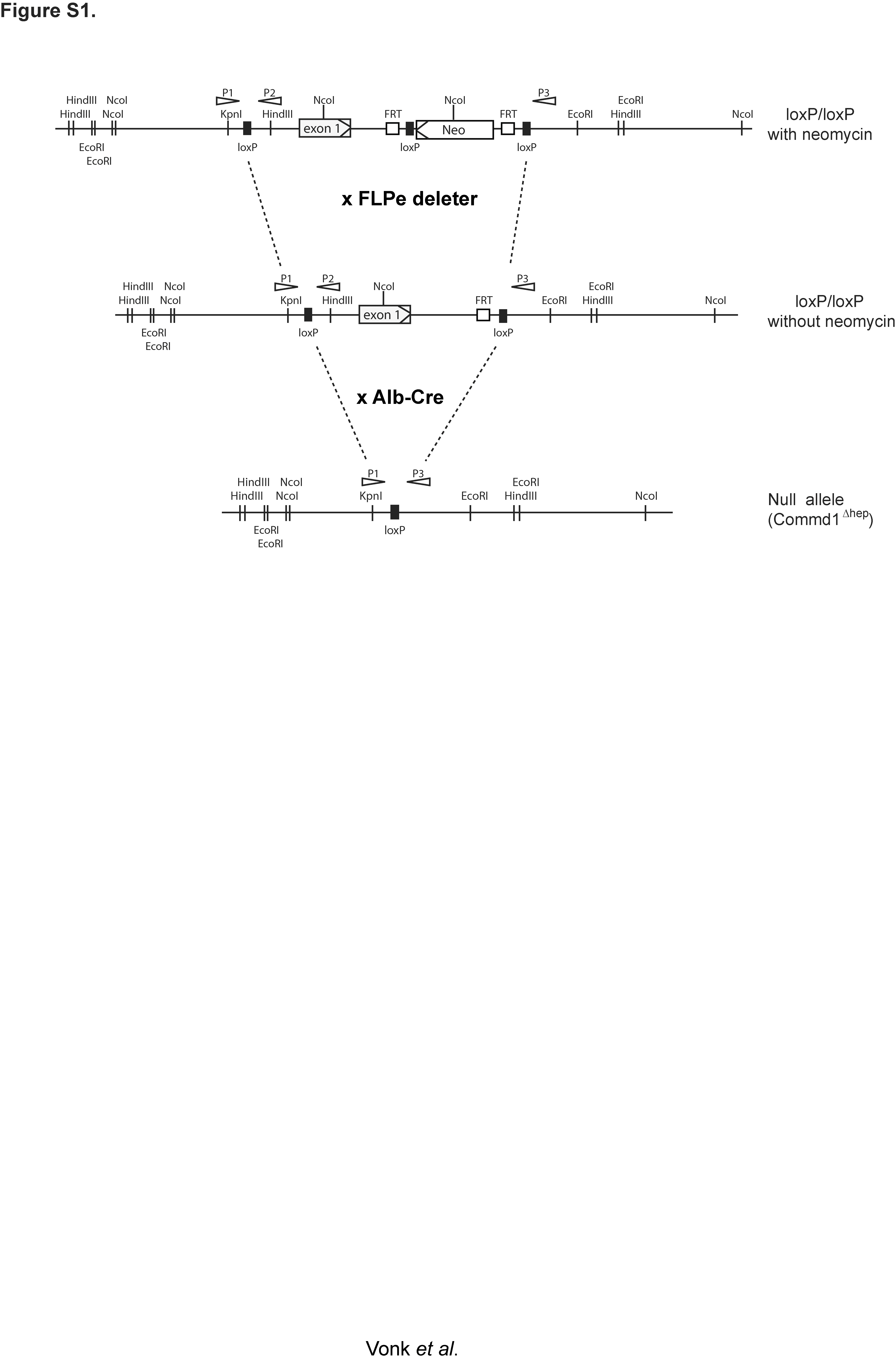

Supplement: Figure S1 — Generation of hepatocyte-specific Commd1 knockout mouse. Schematic representation of the Commd1 gene-targeting strategy used to generate a hepatic specific Commd1 knockout mouse, including a map of the COMMD1 exon1 allele, the targeting vector with loxP sites (solid boxes), FRT sites (open boxes), and neomycin selection gene (Neo). Different restriction sites are indicated and homologous recombination is marked with dotted lines. The neomycin selection cassette was deleted by crossbreed with the FLPe deleter mice, which target the FRT sequences flanking neomycin. Subsequently, hepatocytespecific deletion of Commd1 was accomplished by crossbreed of Commd1 loxP/loxP mice with Alb-Cre mice. This resulted in the generation of Commd1 Δhep mice (null allele). The locations of the PCR primer (P1, P2 and P3) binding sites used for genotyping are shown as open arrows. (TIF) [file pone.0029183.s001.tif]

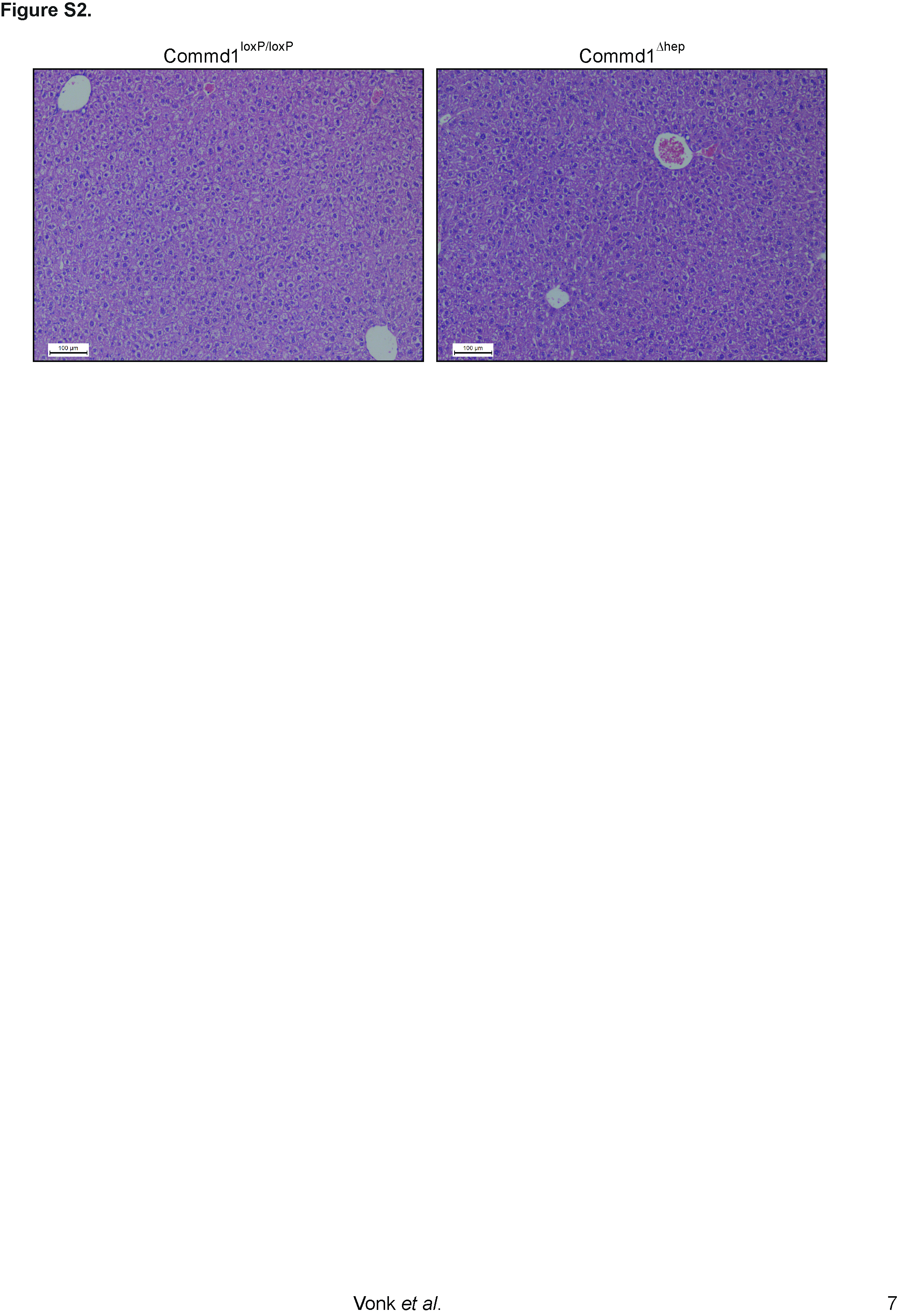

Supplement: Figure S2 — Commd1 Δhep mice do not display any pathological abnormalities relative to Commd1 loxP/loxP mice. Liver sections (4 µm) of Commd1 loxP/loxP and Commd1 Δhep mice fed a copper-enriched diet for 6 weeks were stained with H&E, and analyzed by light microscopy (magnification 10×). (TIF) [file pone.0029183.s002.tif]

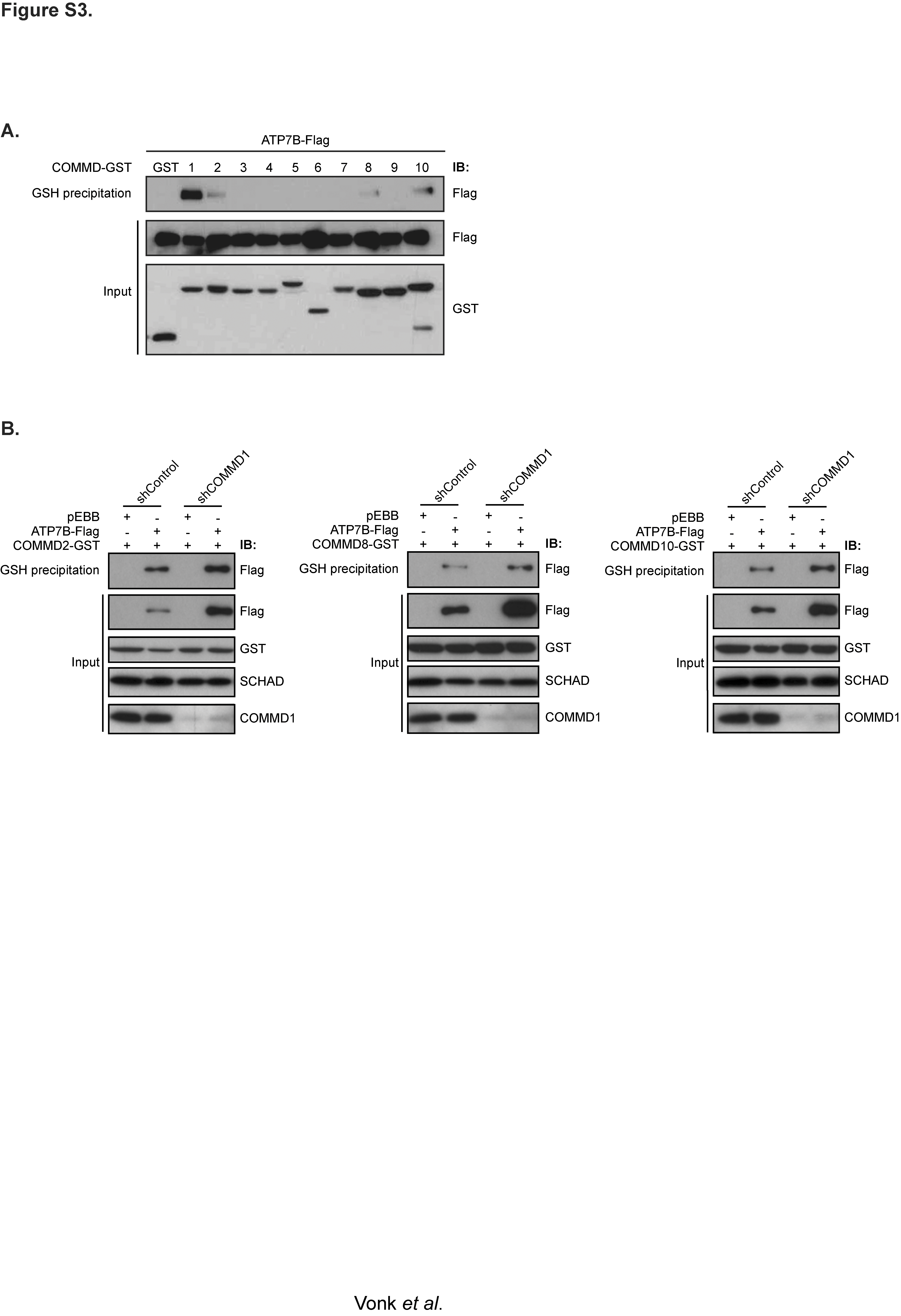

Supplement: Figure S3 — COMMD2, COMMD8 and COMMD10 interact with ATP7B, independently of COMMD1. A.) Gluthatione-sepharose (GSH) precipitation of HEK293T cell lysates transfected with cDNA constructs encoding GST or each of the COMMD proteins fused to GST in combination with ATP7B-Flag. Precipitates were washed and separated by SDS-PAGE and immunoblotted as indicated. Input indicates direct analyses of cell lysates. B.) HEK293T cells expressing a stable knockdown of COMMD1 (shCOMMD1) were transfected with cDNA constructs encoding an empty vector (pEBB) or ATP7B-Flag in combination with either COMMD2, COMMD8, or COMMD10 as GST fusion proteins as indicated. HEK293T cells stably transfected with an empty shRNA vector was used as a negative control (shControl). GSH precipitation and immunoblot analysis was performed as described under S3A. Equal loading was confirmed by immunoblotting for SCHAD. (TIF) [file pone.0029183.s003.tif]
