# Supplementary material for: Liver-Specific Commd1 Knockout Mice Are Susceptible to Hepatic Copper Accumulation
Source: PLoS One. 2011 Dec 22;6(12):e29183. doi: 10.1371/journal.pone.0029183 (PMC3245254; doi:10.1371/journal.pone.0029183)
Supplement: Table S1 — Biological parameters of Commd1loxP/loxP and Commd1Δhep mice fed a standard diet. (PDF) [file pone.0029183.s004.pdf]

Table S1. Biological parameters of Commd1$^{loxP/loxP}$ and Commd1$^{\Delta hep}$ mice fed a standard diet

**Age** (wks)	**6**		**9**		**12**		**34**		**46**		**58**	
	Commd1$^{loxP/loxP}$	*Commd1*$^{\Delta hep}$	*Commd1*$^{loxP/loxP}$	*Commd1*$^{\Delta hep}$	*Commd1*$^{loxP/loxP}$	*Commd1*$^{\Delta hep}$	*Commd1*$^{loxP/loxP}$	*Commd1*$^{\Delta hep}$	*Commd1*$^{loxP/loxP}$	*Commd1*$^{\Delta hep}$	*Commd1*$^{loxP/loxP}$	*Commd1*$^{\Delta hep}$
**Total body weight** (g)	19.7 ± 1.9	18.2 ± 3.2	23.6 ± 2.4	22.5 ± 3.3	24.3 ± 3.1	23.8 ± 3.8	32.5 ± 4.9	33.8 ± 9.0	34.8 ± 6.1	31.1 ± 5.1	37.4 ± 9.5	35.0 ± 8.2
**Liver weight** (mg)	1.04 ± 0.22	0.93 ± 0.19	1.18 ± 0.15	1.15 ± 0.16	1.16 ± 0.12	1.19 ± 0.18	1.43 ± 0.17	1.60 ± 0.58	1.33 ± 0.39	1.29 ± 0.23	1.63 ± 0.59	1.48 ± 0.36
**Hepatic Cu conc** (µg/g)	13.7 ± 2.0	46.2 ± 9.9*	22.6 ± 7.9	29.0 ± 1.1	11.8 ± 6.3	19.2 ± 6.7	10.4 ± 3.0	13.9 ± 1.7	15.1 ± 2.0	16.2 ± 5.7	11.2 ± 1.0	14.8 ± 1.1
**Ceruloplasmin** (U/ml)	2.0 ± 1.44	2.8 ± 0.5	2.7 ± 0.4	2.9 ± 0.9	2.3 ± 1.5	3.4 ± 1.0	2.9 ± 0.8	2.7 ± 0.9	2.4 ± 1.7	2.1 ± 0.8	5.9 ± 1.9	5.2 ± 2.7
**GOT** (U/L)	64.8 ± 8.1	88.7 ± 18.2	66.1 ± 8.7	72.7 ± 13.8	76.4 ± 11.2	80.9 ± 10.4	85.3 ± 15.8	97.6 ± 23.5	62.5 ± 7.6	79.8 ± 16.8	82.8 ± 13.9	56.6 ± 11.7
**GPT** (U/L)	31.1 ± 8.5	46.5 ± 19.5	30.0 ± 5.8	39.4 ± 6.6	37.9 ± 12.1	46.8 ± 11.8	37.1 ± 7.2	42.6 ± 14.6	25.6 ± 8.8	40.3 ± 7.8	49.2 ± 9.8	44.5 ± 17.1

* indicates significantly different values compared to C*ommd1*$^{loxP/loxP}$ mice (* $p < 0.05$). n = 5 - 8
