# Supplementary material for: Liver-Specific Commd1 Knockout Mice Are Susceptible to Hepatic Copper Accumulation
Source: PLoS One. 2011 Dec 22;6(12):e29183. doi: 10.1371/journal.pone.0029183 (PMC3245254; doi:10.1371/journal.pone.0029183)
Supplement: Table S2 — Biological parameters of Commd1loxP/loxP and Commd1Δhep mice fed a high Cu diet, starting at an age of 6 weeks. (PDF) [file pone.0029183.s005.pdf]

Table S2. Biological parameters of Commd1$^{loxP/loxP}$ and Commd1$^{\Delta hep}$ mice fed a high Cu diet, starting at an age of 6 weeks

**High Cu diet** (wks)	**3**		**6**		**28**		**40**		**52**	
	Commd1$^{loxP/loxP}$	*Commd1*$^{\Delta hep}$	*Commd1*$^{loxP/loxP}$	*Commd1*$^{\Delta hep}$	*Commd1*$^{loxP/loxP}$	*Commd1*$^{\Delta hep}$	*Commd1*$^{loxP/loxP}$	*Commd1*$^{\Delta hep}$	*Commd1*$^{loxP/loxP}$	*Commd1*$^{\Delta hep}$
**Total body weight** (g)	25.6 ± 1.7	21.3 ± 2.8	22.9 ± 2.8	22.8 ± 4.5	27.4 ± 4.9	31.0 ± 8.1	30.9 ± 8.0	30.3 ± 8.6	29.4 ± 7.4	38.6 ± 5.3
**Liver weight** (mg)	1.14 ± 0.2	1.01 ± 0.16	1.06 ± 0.24	1.09 ± 0.38	1.12 ± 0.23	1.39 ± 0.43	1.31 ± 0.49	1.36 ± 0.6	1.21 ± 0.31	1.76 ± 0.2
**Hepatic Cu conc** (µg/g)	25.8 ± 10.7	195.8 ± 58.9	28.5 ± 6.5	338.3 ± 82.4***	9.2 ± 3.0	265.2 ± 69.9****	12.9 ± 3.9	291.9 ± 157.2*	16.4 ± 3.8	164.2 ± 75.9***
**Ceruloplasmin** (U/ml)	2.13 ± 0.3	2.2 ± 1.1	3.6 ± 2.3	3.5 ± 0.9	1.7 ± 0.8	2.8 ± 0.7*	3.7 ± 1.4	5.2 ± 1.4*	4.8 ± 2.4	8.2 ± 1.6**
**GOT** (U/L)	79.5 ± 10.3	100.5 ± 23.9	91.6 ± 17.2	72.3 ± 14.2	78.3 ± 12.8	95.1 ± 20.0	85.6 ± 19.7	89.6 ± 16.1	69.7 ± 11.0	58.5 ± 3.7
**GPT** (U/L)	37.1 ± 13.3	47.8 ± 20.1	39.2 ± 11.7	38.2 ± 19.5	28.5 ± 9.2	29.7 ± 17.4	52.6 ± 11.4	43.9 ± 12.1	36.8 ± 6.8	24.2 ± 11.8

*, **, ***, and **** indicate significantly different values compared to C*ommd1*$^{loxP/loxP}$ mice (* $p < 0.05$, ** $p < 0.01$, *** $p < 0.005$, **** $p < 0.0005$). n = 5 - 8
