# Supplementary material for: Liver-Specific Commd1 Knockout Mice Are Susceptible to Hepatic Copper Accumulation
Source: PLoS One. 2011 Dec 22;6(12):e29183. doi: 10.1371/journal.pone.0029183 (PMC3245254; doi:10.1371/journal.pone.0029183)
Supplement: Table S3 — Oligonucleotide sequences used for genotyping mice. (DOC) [file pone.0029183.s006.doc]

**Table S3.** Oligonucleotide sequences used for genotyping mice

| **No.** | **Name** | **primer sequence (5’-> 3’)** |
| --- | --- | --- |
| **P1** | loxP Forward | TGTGAGCTGATTGGGTGTG |
| **P2** | loxP Reverse | GGAAAATGGTATAATAGACTATG |
| **P3** | AT4 Reverse | CACCTGTTATGTTGCCCTC |
