# Supplementary material for: Liver-Specific Commd1 Knockout Mice Are Susceptible to Hepatic Copper Accumulation
Source: PLoS One. 2011 Dec 22;6(12):e29183. doi: 10.1371/journal.pone.0029183 (PMC3245254; doi:10.1371/journal.pone.0029183)
Supplement: Data S1 — Supplementary Materials and Methods . (DOC) [file pone.0029183.s007.doc]

**Supplementary data**

**Supplementary Materials and Methods**

*Generation of hepatocyte-specific Commd1 knockout mouse model*

A *Commd1* targeting construct in which exon 1 of *Commd1* was flanked by loxP recombination sites followed by a neomycin selection cassette was designed (Figure S1). The targeting vector was electroporated into 129/SvEv embryonic stem (ES) cells, and screened for homologous recombination by long-range PCR. Two clones positive for homologous recombination were amplified and used to generate chimeric mice. F1 heterozygote mice derived from each clone were crossed with Flp germ-line deleter strain (FLPe deleter strain (B6;SJL-Tg(ACTFLPe)9205Dym/J); The Jackson Laboratory, Bar Harbor, Me, USA) in order to remove the neomycin resistance cassette (Figure S1). Mice lacking the neomycin resistance cassette (*Commd1*P^loxP/loxP^P) were kept on a mixed 129/SvEv x C57Bl/6 genetic background. Hepatic Commd1 deficiency (*Commd1*^Δhep^) was accomplished using Albumine-Cre (Alb-Cre) transgenic mice, which were also obtained from the Jackson Laboratory (strain name: B6.Cg-Tg(Alb-cre)21Mgn/J) (Figure S1). The phenotype discussed in this study was observed in two *Commd1*P^Δhep^P mouse lines, which were generated from independent ES clones.

*DNA isolation and analysis*

DNA was isolated from mouse liver by homogenization in SE buffer (75 mM NaCl, 25 mM EDTA; pH 8.0) prior to overnight incubation at 55°C in Proteinase K lysis buffer (10 mg/ml Prot. K, 10% SDS). DNA was extracted by means of phenol/chlorophorm/isoamyl ethanol (25:24:1), and precipitated with 3 M NaAc/HAc; pH 5.2 in absolute ethanol.

*Histology*

Mouse livers were fixated overnight in 4% paraformaldehyde in PBS, dehydrated in 70% ethanol and embedded in paraffin. 4 µm thick sections were stained with hematoxylin and eosine (H&E). Stained sections were analyzed and captured using a Leica DM3000 microscope and Leica Software.

*Cell culture and transfections*

Human embryonic kidney 293T (HEK293T) cells were obtained from ATCC (Manassas, VA, USA) and cultured in high-glucose Dulbeco’s modified Eagle’s medium GlutaMAXP^TM^P (4.5 g/L D-glucose and pyruvate; Invitrogen Life Technologies Corporation, Carlsbad, CA, USA) supplemented with 10% fetal bovine serum (FBS), L-glutamine, penicillin and streptomycin at 37°C in 5% CO_2_. Monoclonal HEK293T, stably transfected with pSUPER-RETRO vector (shControl) or a plasmid encoding short hairpin RNA (shRNA) targeting COMMD1 mRNA sequence (shCOMMD1) were described previously [1] and maintained in HEK293T medium supplemented with 1 μg/μl puromycin dihydrochloride (Sigma-Aldrich, St. Louis, MO, USA),

*Constructs*

pEBB-COMMD-GST and pEBB-ATP7B-Flag constructs were described previously [2,3].

*Reagents*

The following antibodies were used for immunoblotting: rabbit-anti-COMMD1 antiserum [4], polyclonal mouse-anti-Flag M2 HRP conjugated (Sigma-Aldrich), polyclonal rabbit-anti-GST (Santa Cruz Biotechnology, Santa Cruz, CA, USA), and short-chain L-3-hydroxyaceyl-CoA dehydrogenase (SCHAD) antiserum [4].

**Supplementary references**

1. van de Sluis B, Muller P, Duran K, Chen A, Groot AJ, et al. (2007) Increased activity of hypoxia-inducible factor 1 is associated with early embryonic lethality in Commd1 null mice. Mol Cell Biol 27: 4142-4156.

2. Burstein E, Hoberg JE, Wilkinson AS, Rumble JM, Csomos RA, et al. (2005) COMMD proteins, a novel family of structural and functional homologs of MURR1. J Biol Chem 280: 22222-22232.

3. de Bie P, van de Sluis B, Burstein E, van de Berghe PV, Muller P, et al. (2007) Distinct Wilson's disease mutations in ATP7B are associated with enhanced binding to COMMD1 and reduced stability of ATP7B. Gastroenterology 133: 1316-1326.

4. Klomp AE, van de Sluis B, Klomp LW, Wijmenga C (2003) The ubiquitously expressed MURR1 protein is absent in canine copper toxicosis. J Hepatol 39: 703-709.

**Supplementary Table**

**Table S1.** Oligonucleotide sequences used for genotyping mice

| **No.** | **Name** | **primer sequence (5’-> 3’)** |
| --- | --- | --- |
| **P1** | loxP Forward | TGTGAGCTGATTGGGTGTG |
| **P2** | loxP Reverse | GGAAAATGGTATAATAGACTATG |
| **P3** | AT4 Reverse | CACCTGTTATGTTGCCCTC |
